# Experimental investigation of four-point bending of thin walled open section steel beam loaded and set in the shear center

**DOI:** 10.1038/s41598-022-10035-z

**Published:** 2022-05-04

**Authors:** Maciej Obst, Piotr Wasilewicz, Jarosław Adamiec

**Affiliations:** 1 Division of Strenght of Materials and Structures, Poznan University of Technology, Poznan, Poland; 2 Institute of Machine Design, Poznan University of Technology, Poznan, Poland

**Keywords:** Mechanical engineering, Mechanical properties

## Abstract

Thin walled cold formed structures were, and still are, very popular structural elements used in mechanical engineering. Modern technology and the progress in materials engineering allow to fabricate various shapes of thin walled cold formed components. Therefore, combinations between technology possibilities, material properties, loads and engineering requirements are wide and unlimited for thin walled components. The aim of this paper is to perform an experimental study of cold formed C-channel steel beams under four-points bending loads based on the global and the local buckling phenomena. A test rig was developed with a specially designed support system to subject thin-walled steel beams to a four-point bending load, where the support and loads were applied to the shear center of the open section steel beam. It is shown that it is not possible to completely eliminate the torque load on a thin-walled beam with an open section where load and support are applied in the shear center because investigated beams weren’t ideal made. Thin walled open section beams are very sensitive for boundary conditions and geometrical accuracy. The force of gravity is also working. The presented research methodology can be improved and do test with other open section thin walled beams.

## Introduction

The advantages of thin walled cold formed structures over classical metallurgical sections are such that constructions with similar durability properties are much lighter. Additionally, cold shaped thin walled components have less limitations considering the forming process. Last year’s technological development gave possibilities for almost any cold shaping of steel details. Analytical methods with open section thin walled rod and beams were originate from Vlasow (1940) theory, where small displacements assumptions and linear properties between stress and strains form base for formulated mathematical dependencies. Thin walled open section beam theory is based on long shell and plates theory assumptions, where structural stiffness, global and local stability have the key role during design. Introduction for theoretical stability of bars, shells, plates and other structures are described by Timoshenko and Gere^1^. Nowadays, there are many research papers, where the strength and the stability of thin walled structures are of interest for engineers and scientists.

The stability of thin-walled beams with open section was studied by Magnucki et al. and Magnucka-Blandzi and Zając^2^. Magnucki et al.^3^ in their monograph described stability issues in applied mechanics problems. They described the stability of basic structural models such as bars, beams and connected systems of structural bars. They thoroughly presented the stability of thin rectangular or circular plates and rotating shells. Their work includes a chapter on the stability of thin-walled beams with open sections. The authors applied energy-based methods to analytical studies, presenting a practical problem for steel tank issues. They presented practical examples of the application of the finite element method and modern stability testing experiments. The topic of stability of thin-walled structure was also analysed by Anbarasu^4, 5^. He presented results of cold-formed steel thin walled lipped channel buckling behavior. Experimental and computational research method were developed. Investigated buckling modes interaction such as local, distortional, lateral, torsional was subjected to a numerical model. Ultimate resistance and adequate bending moment was estimated. Anbarasu^5^ in his research ignored residual stresses and an applied elastic-perfect plastic material model without strain hardening. Proposed analytical formula, where interactions between buckling modes was estimated, can be an interesting design tool for engineers.

Anbarasu at al.^6^ and Dar at al.^7^ presented the results of an experimental study of thin-walled steel beams constructed from channels, where the flanges have a bend in the form of a band—the so-called Lipped channels, positioned with webs relative to each other with a certain distance and steel plates connected to the channel flanges by self-tapping screws with washers. The tested beams were subjected to 3- and 4-point bending. The tested beams consisting of steel channels and plate flanges were a symmetrical system, where both the direction of reaction forces and static load intersected the center of gravity of the section and the center of transverse forces. Steel plates were welded to the ends of the beams to prevent warping of the section during testing. The authors pointed out the local buckling which characterizes the thin-walled structures designated as CFS. In another paper^8^, the authors presented the results of a series of experimental tests of rectangular CFS composite beams with compression flange, showing higher stiffness of these structures compared to conventional solutions. Another experimental study^9^ of thin-walled CFS structures that were reinforced with angle sections to increase the stiffness of the structure and the load-bearing capacity of the beam. Beams with both open and closed sections in four-point bending configuration were investigated for different stiffening systems. The paper points out that proper selection of stiffening elements increases the load carrying capacity and stiffness of a beam made with CFS technology from 85 to 100%. In the next paper, Ambaransu^10^ presented the results of a simulation study of beams with closed sections made with CFS technology. Referring to the literature data from experimental studies, he carried out the validation of the simulation model in Abaqus software. The tests were carried out for different cross-sectional shapes of the beams and different thicknesses of the insertion elements of the CFS steel beams.

Experimental study of buckling problems for Asymmetric I-Section beams was done by Balasubramanian et al.^11^. Obtained results were verified by the FEM method. Presented experimental methodology was tested for different cross section parts under four point bending by load forces into two pints in the flange plane. Critical load for each tested member was specified. Belingardi et al.^12^ experimentally investigated steel thin walled top-hat box beams, where adhesive joints were applied between connected parts. Three point bending was used under load. Behaviors of the three types of members were investigated. Composite laminate plate was used during the test as a layer between specimen and thin walled member. Interaction between beam buckling modes for four-point bending was developed by Shokouhian et al.^13^ experimentally, analytical and by finite element method. Buckling and post buckling of thin walled slatted aluminum columns subjected to compressive forces was investigated by Ziółkowski et al.^14^. During experimental tests displacement control of compression forces was a new research proposition. Aluminum made specimens used for experimental tests were made geometrically very precise which turns out to be a very important factor in experimental research on thin-walled structures. Mechanical properties of aluminum columns were accurately determined with changes of Poisson ratio estimation as well. During the experiment authors used test strain gauges glued in critical places for designate plastic buckling. As a result of experimental buckling of aluminum columns was material plastic yielding, elastic and inelastic buckling. Authors divided considered specimens depending on their slenderness and buckling mechanism.

Interesting paper by Rusiński et al.^15^ describes thin walled structures stability problems, where steel sheet metal parts of thin walled structure were joined by spot welding. Authors experimentally and computational by the FEM method investigated thin walled, closed section parts under axial compression loads. Weld diameter and the pitch of the weld were controlled for the amount of the energy absorption. All structural elements are fixed by various techniques. Applied connection method, technology and exploitation conditions can have the key role in the view of energy absorption characteristics of static and dynamic stability of thin walled structures. Thin-walled structures have the added practical advantages of dissipating mechanical energy. An experimental study of thin walled cold formed steel beams subjected to the monotonic and cyclic loading was conducted by Calderoni et al.^16^. Dynamic experimental research was based on changes of displacement amplitude. During the monotonic tests the reaction forces and the specimen displacement were measured. Based on the received characteristics of the force and the displacement dependencies, particular phases were separated. What could be observed is a stable state phase, critical force due to local buckling and unstable state due to collapse of the tested section. For the cyclic test it was noted that investigated beams behavior was characterized by progressive reduction of load bearing capacity. Typically,local buckling of flanges lead to destruction of the part.

In the paper of He et al.^17^ thin-walled, open section beams were investigated for buckling problems. Two methods have been used for analysis of three different cross section shape members: semi-analytical finite strip transfer matrix method and transfer matrix method. Obtained results were compared to the finite element method simulation. Authors widely described the evolution of numerical methods and different concepts that were applied in buckling analysis. Investigated members: asymmetric E-section, symmetric I-section and X-section proposed strategies and buckling results are compared and discussed. The effect of the imperfection on perforated thin walled cold formed structure was investigated by Ungureanu et al.^18^. The experimental investigations were supplemented by the authors with numerical simulations using the finite element method. The authors concluded that in order to ensure the stability of thin-walled cold-formed structures, it is necessary to additionally carry out a reliability analysis that can produce results for a given failure probability. An experimental study of the Bauschinger effect in a 1 mm thick thin steel sheet subjected to tensile and four point-bending tests was addressed by Kato et al.^19^. Tensile test was used for residual stress implication in specimens, then wire strain gauges were used during experimental research and bending loads. As a result, stress and residual strain level are what can be important for thin walled structure engineers when dynamic loads or cyclic loading and unloading are exploitation conditions.

It should be noted that the above research represents only a small part of the research that concerns the field of using thin-walled beams with an open section as energy-absorbing elements of safety barriers. Thin-walled beams are also used as energy-absorbing elements in vehicle construction as passive protection. Authors of the paper Vignjevic et al.^20^ presented research results for stability structures problems in vehicle safety. Cold formed thin walled structures are commonly applied in automotive body structures. Modern vehicles body is designed with a focus on vehicle passive safety. During a crash the loads have different directions, intensity and place of application. First stage of structure destruction is linear elastic deformation, then plastic deformation and buckling, material tearing and joints destruction. Author Vignjevic^20^ noticed that the stiffness of the thin walled body structure decreases during impact load. Valuable observation is a limitation of beam theory because thin walled dynamic structure deformation directly depends on material properties, manufacturing process and design. In the paper Vignjevic^20^ focused on energy absorption by thin walled structures such as rectangular beams under uniaxial and biaxial deep bending destruction. Dynamic investigation of thin walled structures seems a necessary area of research.

Authors Obst et al.^21^ studied experimentally and analytically thin-walled steel beams with an open section, the setting points and load points were in a web plane and additional diaphragms were used. In another paper, authors Obst et al.^22^ also presented a stability study of a thin-walled open section beam subjected to four-point bending. Boundary conditions have a key role in stability of thin walled structures. Investigated beams under four-point bending was load and set in center of gravity of beam cross section.

**Figure 1:**
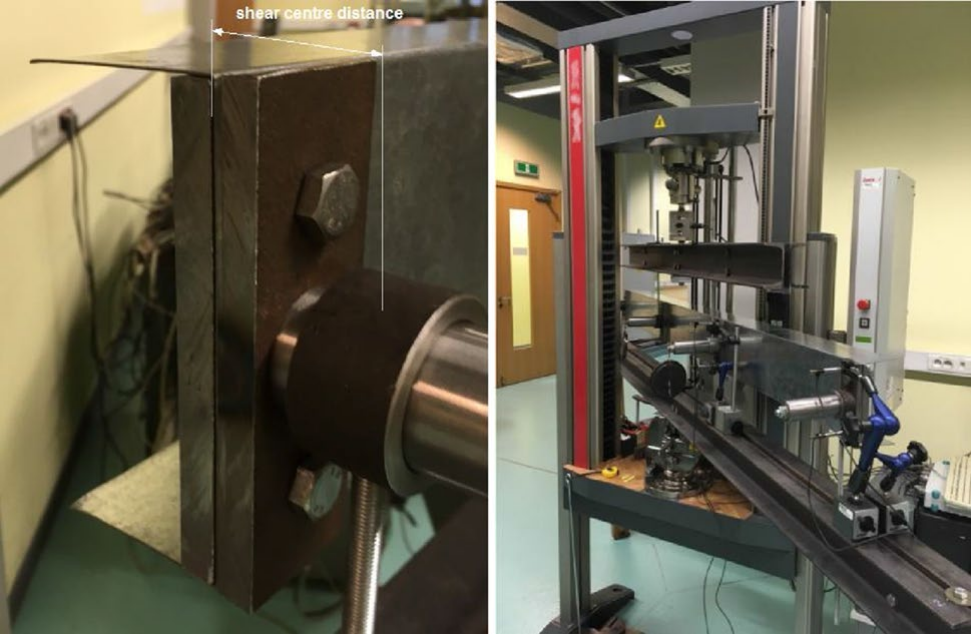
Web stiff steel plates and hinge which allow set and load the beam in shear centre.

**Figure 2:**
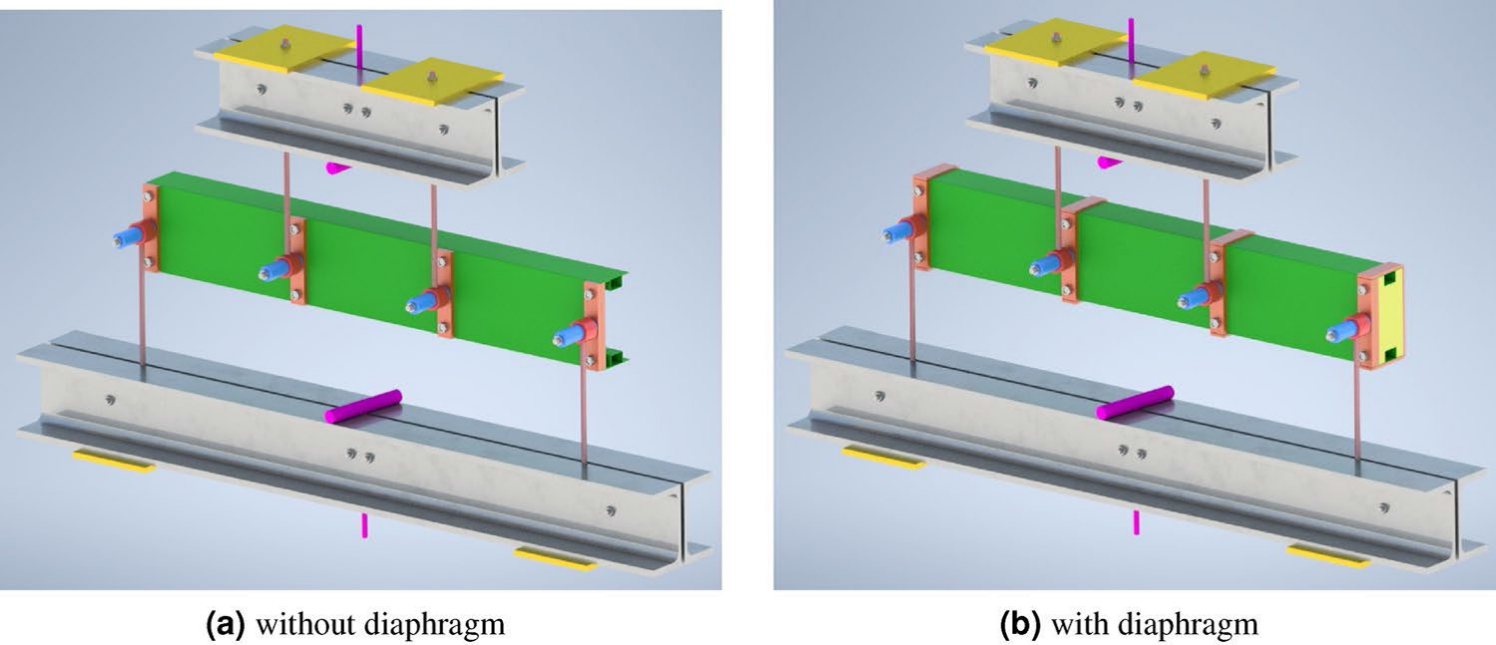
Bending.

In this paper authors presented result of experimental research of three types C-shape open section steel beams which were support and loaded in shear center by special handles. The handles used during experimental research was consisted with: cylindrical pin, ring fitted to pin and bracket bolted to beam web. Distance between center of the ring and beam web determined the location of calculated shear center for a given cross section shape. On the Fig. 1, 2, 3 handles are visible.

**Figure 3:**
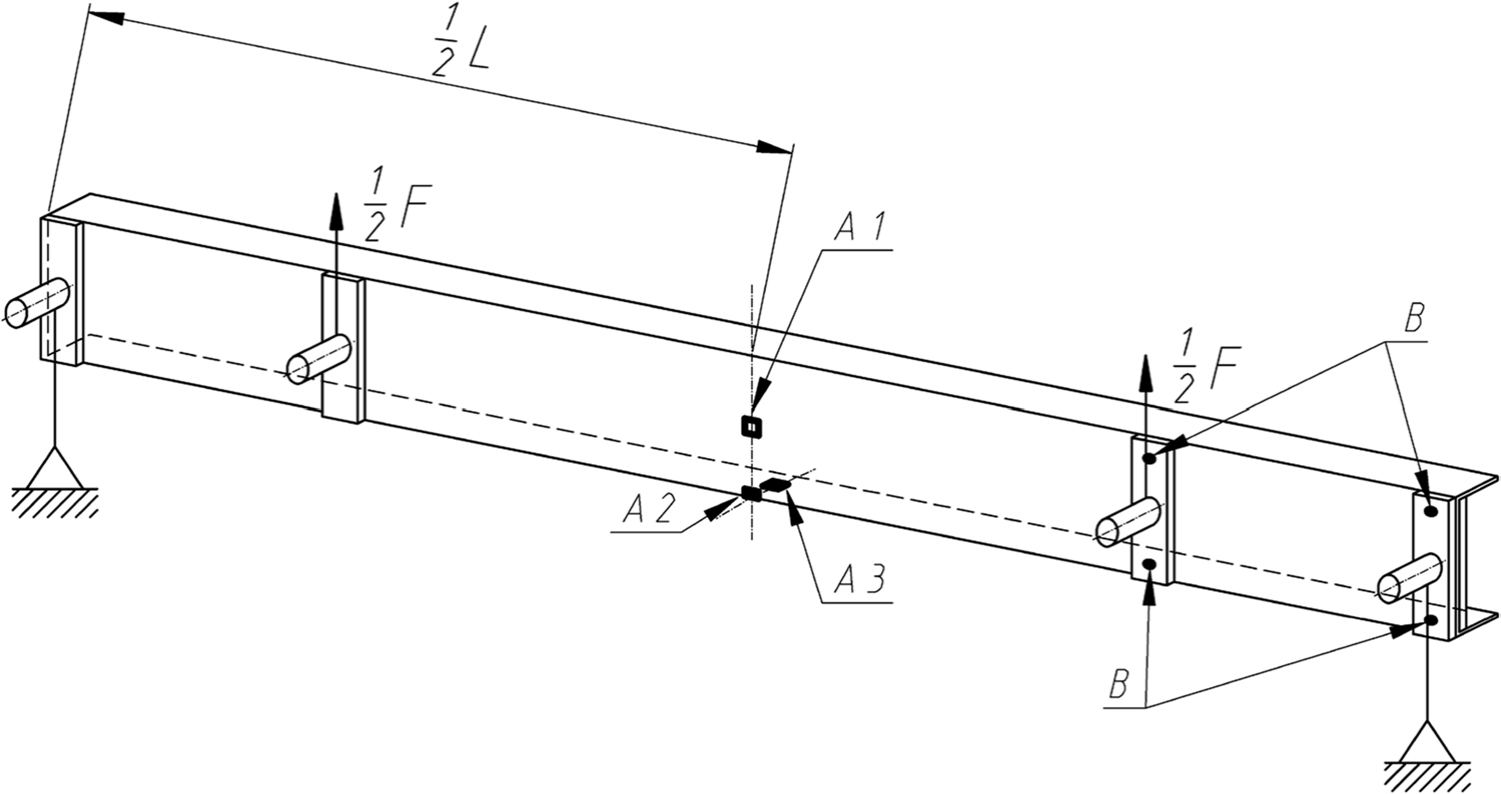
Schematic of the placement of strain gauges (A1, A2, A3) and displacement sensors (B).

## Methods

**Figure 4:**
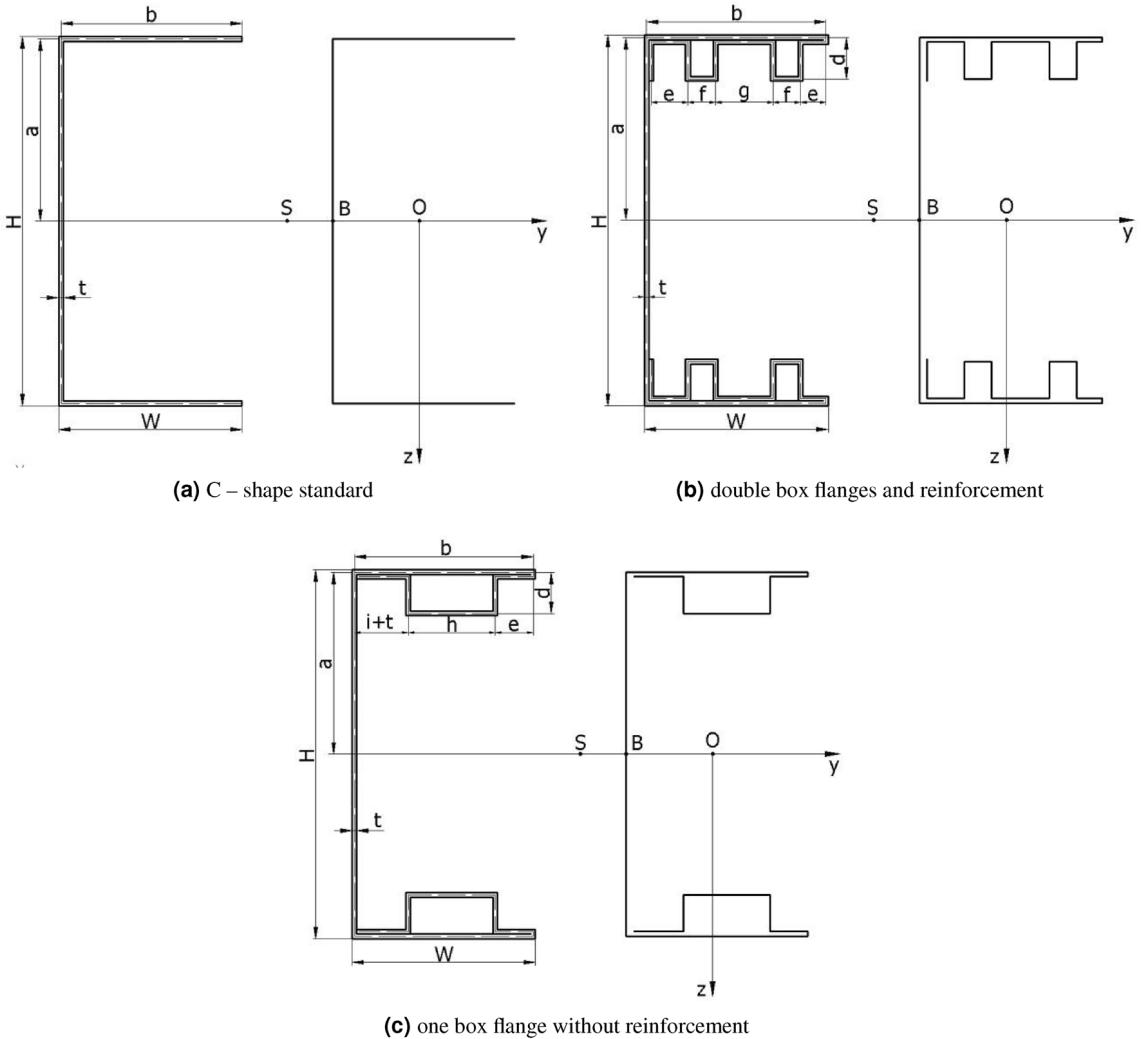
Beam cross section dimensions.

For experimental investigation three different C-shape cross section cold formed steel beams were used, as shown on Fig. 4. During the experiment, the beam specimen was set by a special support system where four points bending was applied. Additionally set points and force points were applied to the shear center of the tested thin walled beam. Experimental research was prepared for beams with the following transverse and longitudinal dimensions (Table 1).

**Table 1:** Beam cross sections dimensions.

Web height	*H*	160 mm
Flange width	$b + t$	80 mm
Wall thickness	*t*	1 mm
Total length	$L_c$	2000 mm
Working length	*L*	600 mm
Distance between supports	$L_0$	1950 mm
Distance between support and concentrated force	$L_s$	675 mm
Bend height	*d*	18 mm
Bend length	*e*	14 mm
Box distance	*f*	18 mm
Box width	*g*	14 mm
Box distance	*h*	39 mm
Bend length	*i*	25 mm

**Figure 5:**
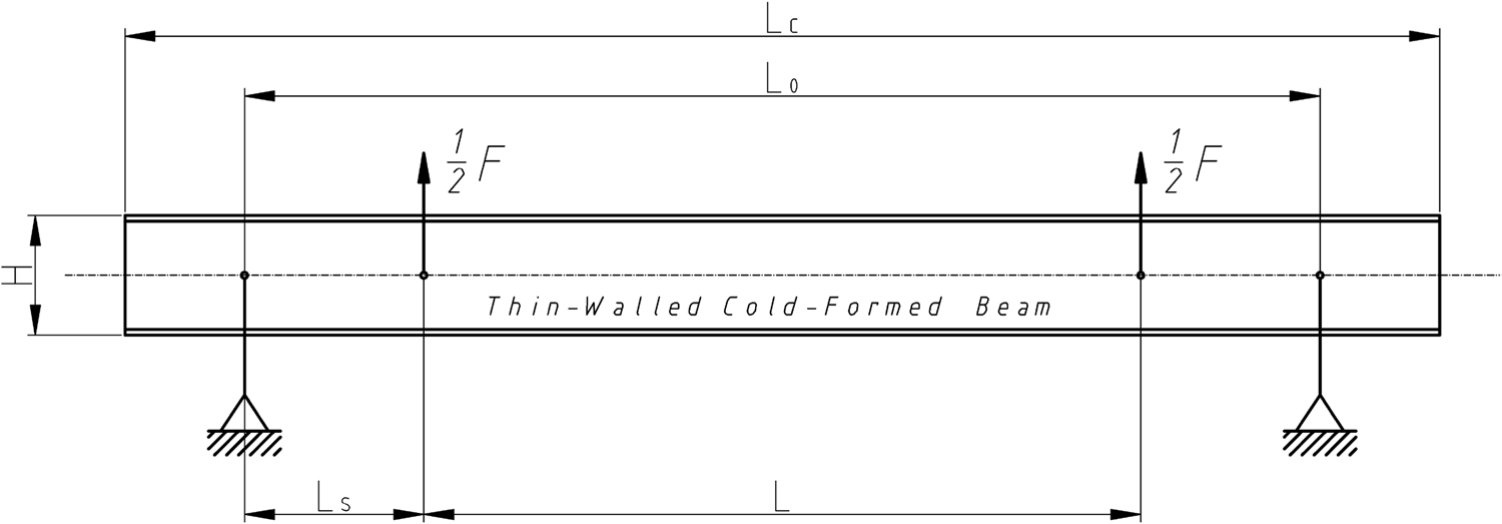
Load scheme and longitudinal dimensions of the beam.

Load scheme of the beam and its dimensions are given in Fig. 5. Setting points and loads points of the specimen beams was a special device mounted to the beam web by stiff steel plates and screws presented on Fig. 1. Simple regulation gave possibilities to lock position in the shear centre of the open section specimen beams. Experimental research was prepared for two setting systems: without and with diaphragm what is shown on Fig. 2.

**Figure 6:**
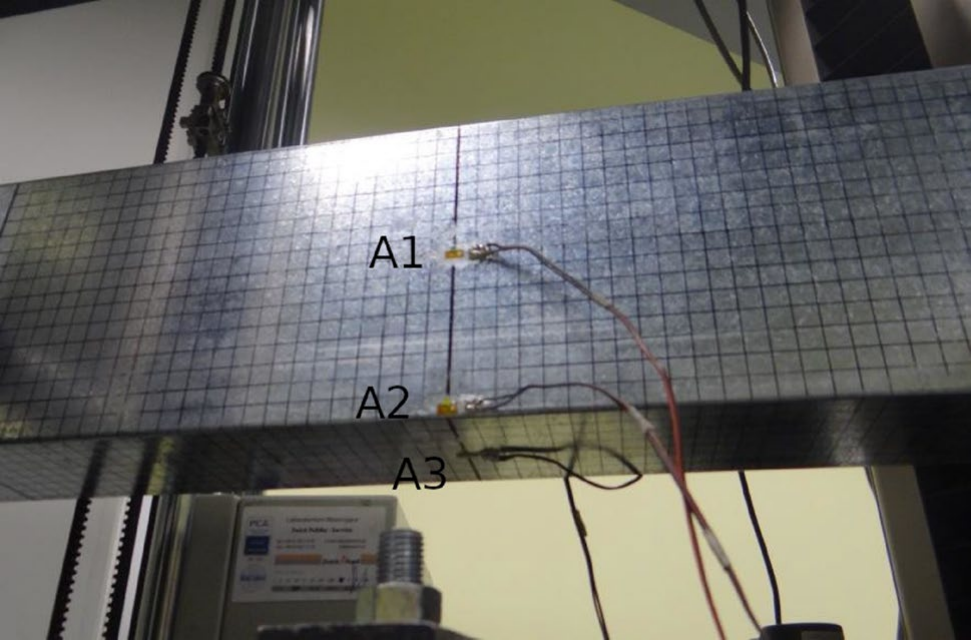
Strain gauges connected to the specimen web and flange.

Experimental tests were prepared and executed on the tensile tension test machine Zwick Z100, equipped with an additional stiff beam system and setting which allowed to realize four points bending of thin walled specimen with shear centre boundary conditions. Each tested specimen beam was equipped with strain gauges connected to the corner and centre of the web (Figs. 3,  6) and to the centre of the beam flange.

**Figure 7:**
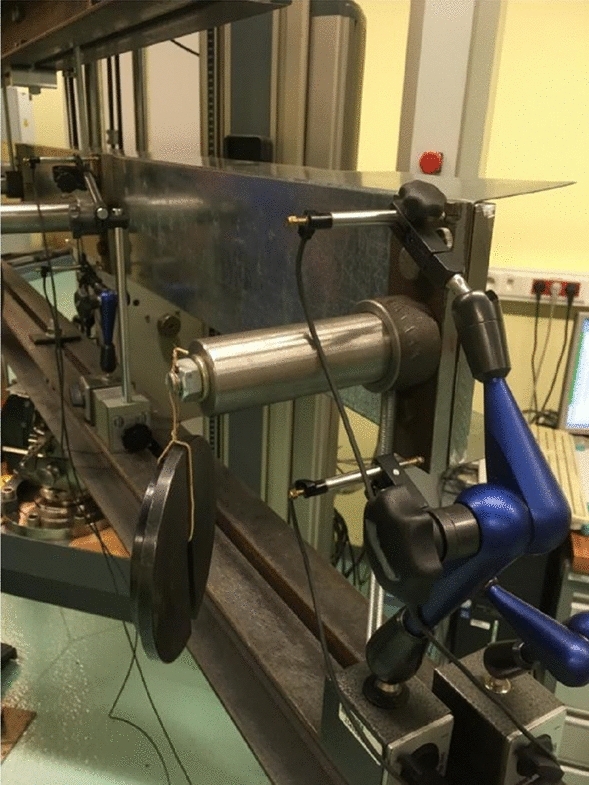
Displacement sensors applied to four points of stiff steel plates.

Additional to the specimen web was installed inductive displacement sensors which measured displacement in four points of the web stiff steel plates corners as shown on the Figs. 3 and 7.

Displacement sensors applied to the specimen web stiff plates were used for analyse rotation of the specimen cross section during load rising. Investigated beams loaded and set in shear centre should be under pure bending moment, which is hard to realize in real experimental conditions.

## Results

**Figure 8:**
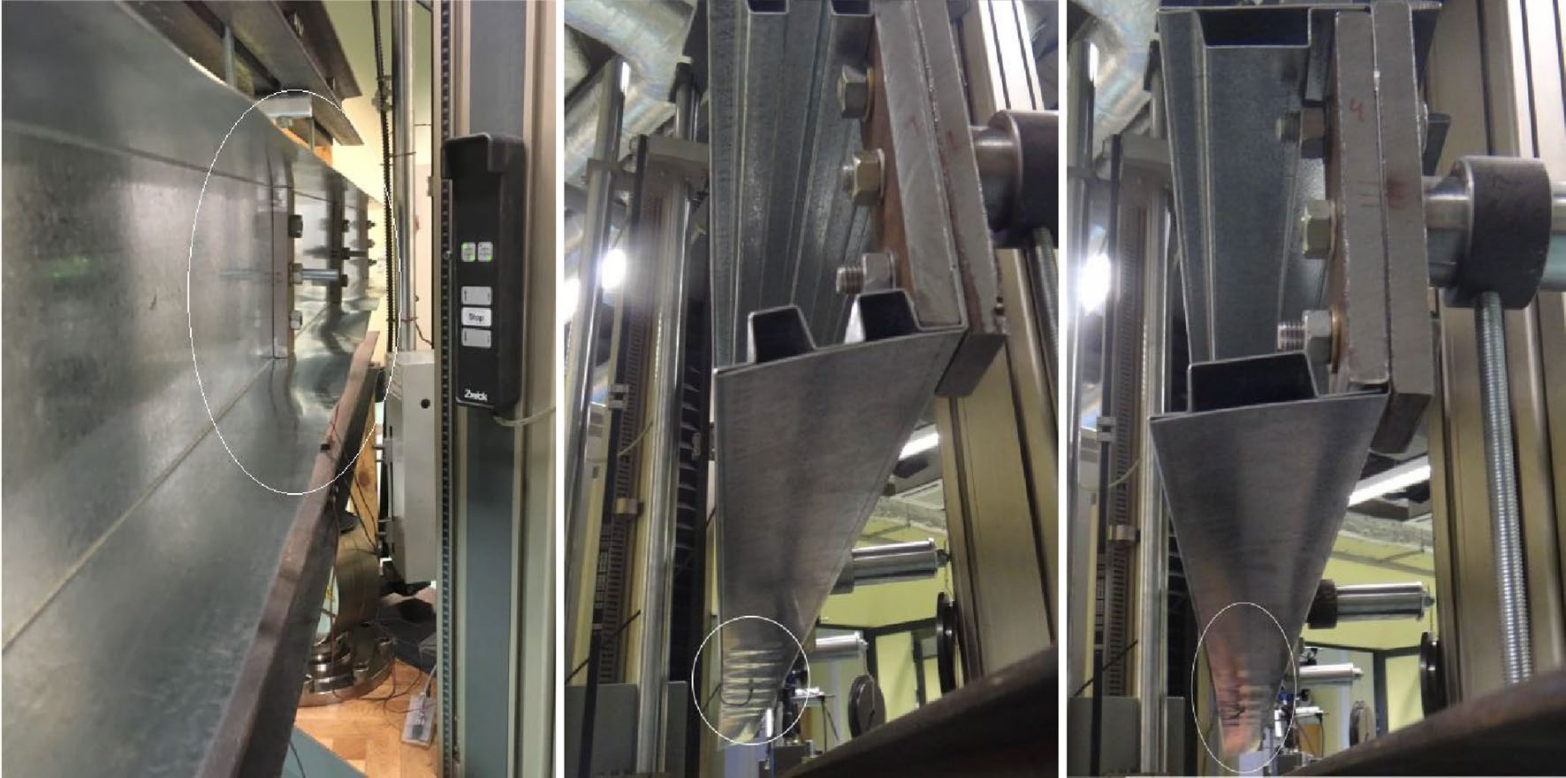
C-Shape no reinforced, double box flange and one box beam after loss of stability.

**Figure 9:**
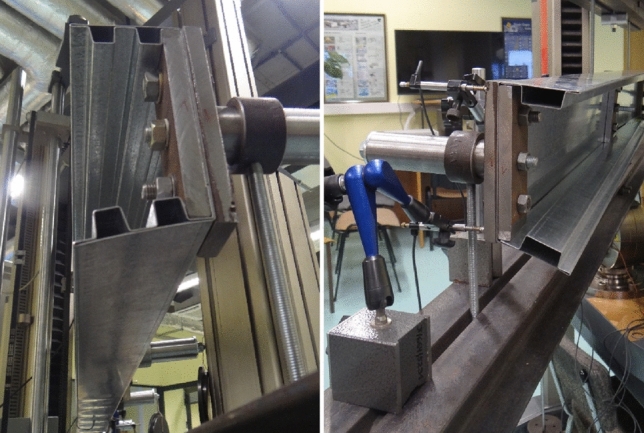
Deformation of cross section beam during experiment of four-point bending.

During the experimental test, three different cross section shape beams were investigated. Tensile machine traverse speed was $5\ {\text{mm/min}}$ and initial force equal $0,5\ {\text{kN}}$. All specimens were supported and load in the shear center of the specimen by special handle. The distance of the shear center from the web was determined analytically for a given cross-section of the thin-walled beam. The position of the shear center was measured with an accuracy of $0,1\ {\text{mm}}$. The error in the physical positioning of the shear center exists and results from the accuracy of the measurement of the distance from the web as well as the accuracy of the beam manufacturing, e.g. longitudinal straightness of the beam. Tested specimen was under four points bending as shown at Fig. 2a. Static increasing loads applied by tension test machine Zwick Z100 from zero to buckling point until beams collapsed. It was observed, initiation of the buckling and destruction by bending where lower flanges start to wrinkle as showed in Fig. 8. Torsion of the beams was observed during the test and is shown in Fig. 9.

**Figure 10:**
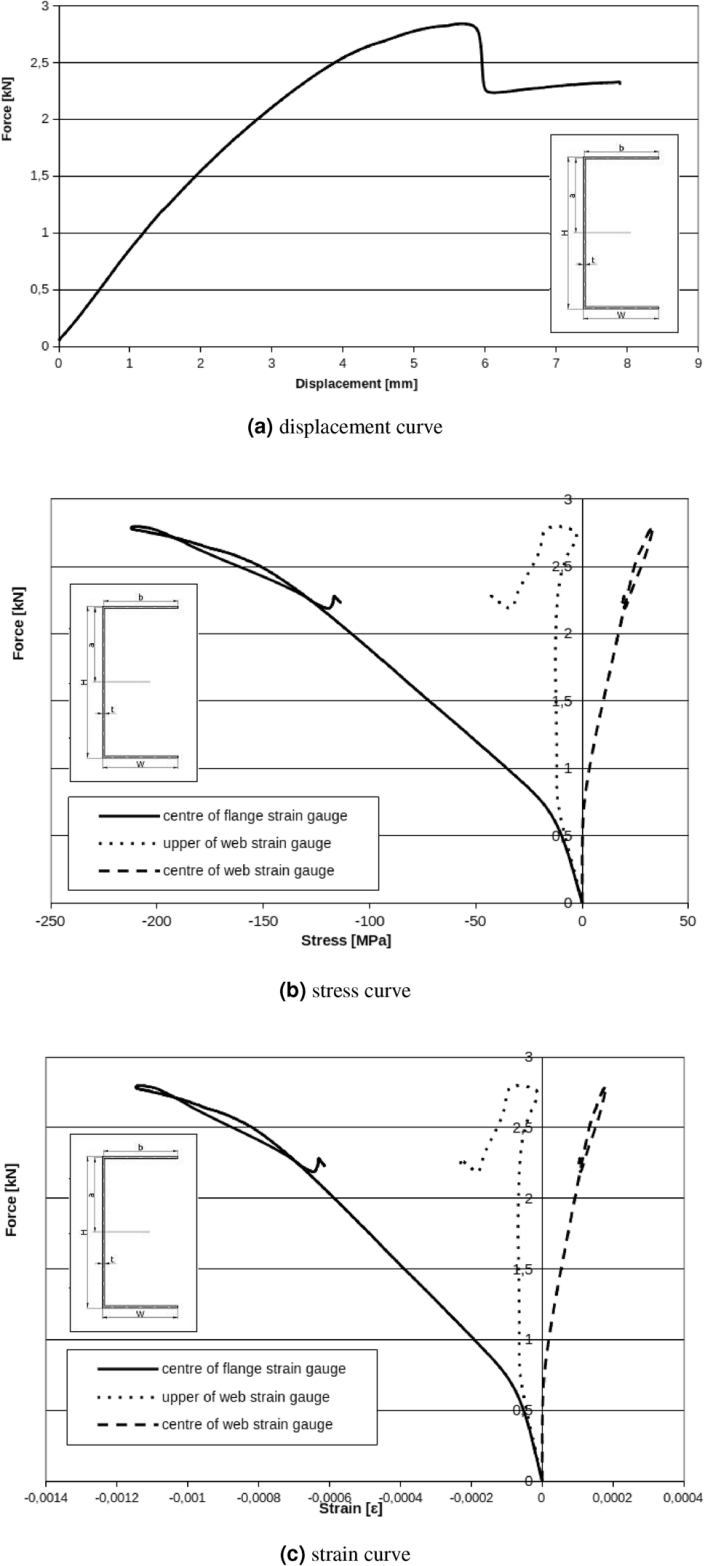
C-Shape no reinforced member.

The experimental research investigated three different cross section thin-walled beams that were subject to the pure bending. During static tests by applied four points bending, shear centre loads and sets, pure bending should be realized. Unfortunately, it was not possible to get ideal pure bending. During monotonically load growth it was possible to observe a little torsional distortion of the beams. Steel mechanical properties of the beam specimens were determined during tensile tests of flat specimens cut from material of the beams. The measured properties were: Young modulus $E = 185\ {\text{GPa}}$, Poisson ratio $\nu = 0.3$, Yield point $R_{eH} = 330\ {\text{MPa}}$, Tensile strength $R_m = 380\ {\text{MPa}}$. On the base of experimental test following results were to work out (Fig. 10).

**Figure 11:**
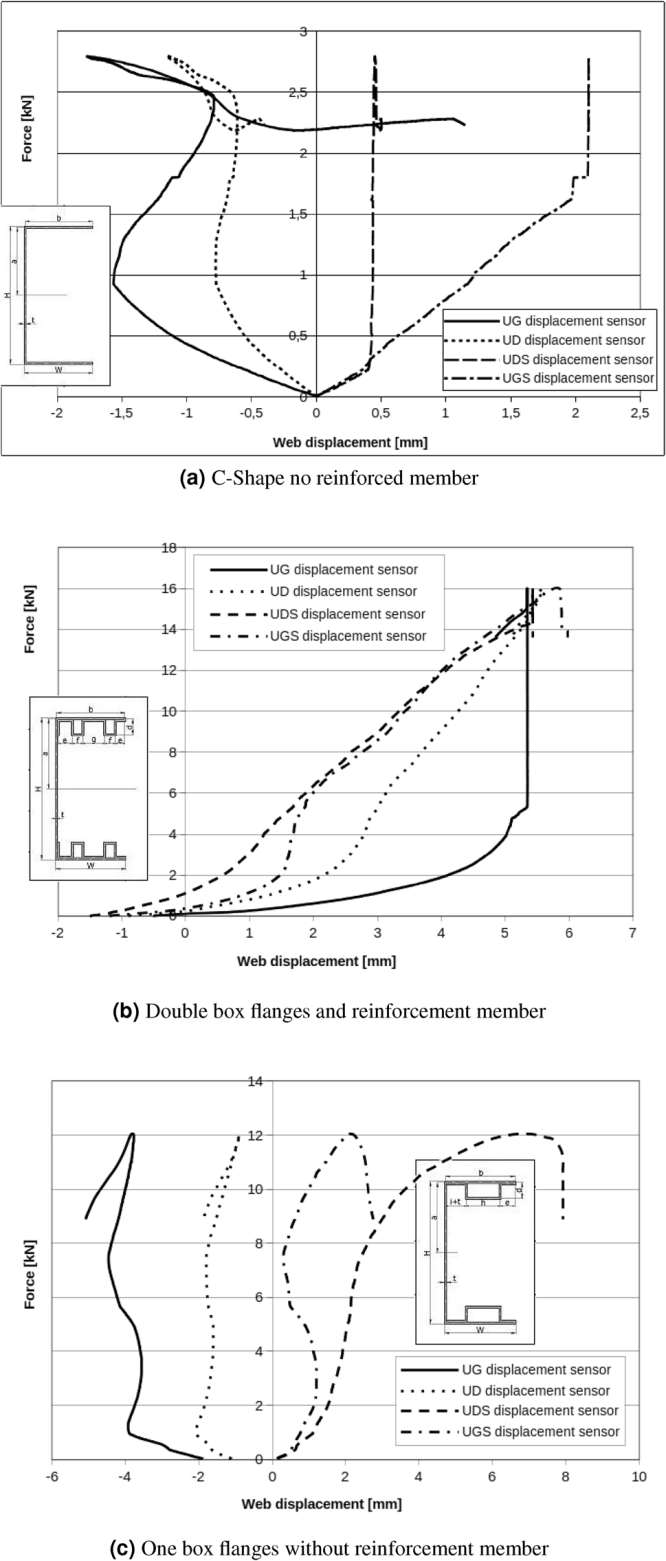
Web displacement points. UG – displacement of the upper web point on the fixed stiff steel plate, UD – displacement of the lower web point on the fixed stiff steel plate, UGS – displacement of the upper web point on the movable stiff steel plate, UDS – displacement of the lower web point on the movable stiff steel plate.

In Fig. 11a the relation between displacement of measuring points can be observed. Presented results can be interpreted as beam cross section rotation around longitudinal x axis. C-shape cross section beam under applied load rotate even under small loads. Cause of that situation are technological and material imperfections. Applied handles are the base of the featured situation. Experimental methodology also can be the cause of the described problem. Based on experimental characteristics Fig. 10 can be find critical force for local and global buckling respectively: $F_{Lcr}=0.6\ {\text{kN}}$, $F_{Gcr}=2.6\ {\text{kN}}$. Local and global buckling on the charts is interpreted as the beginning of characteristic nonlinearity (Fig. 11b).

**Figure 12:**
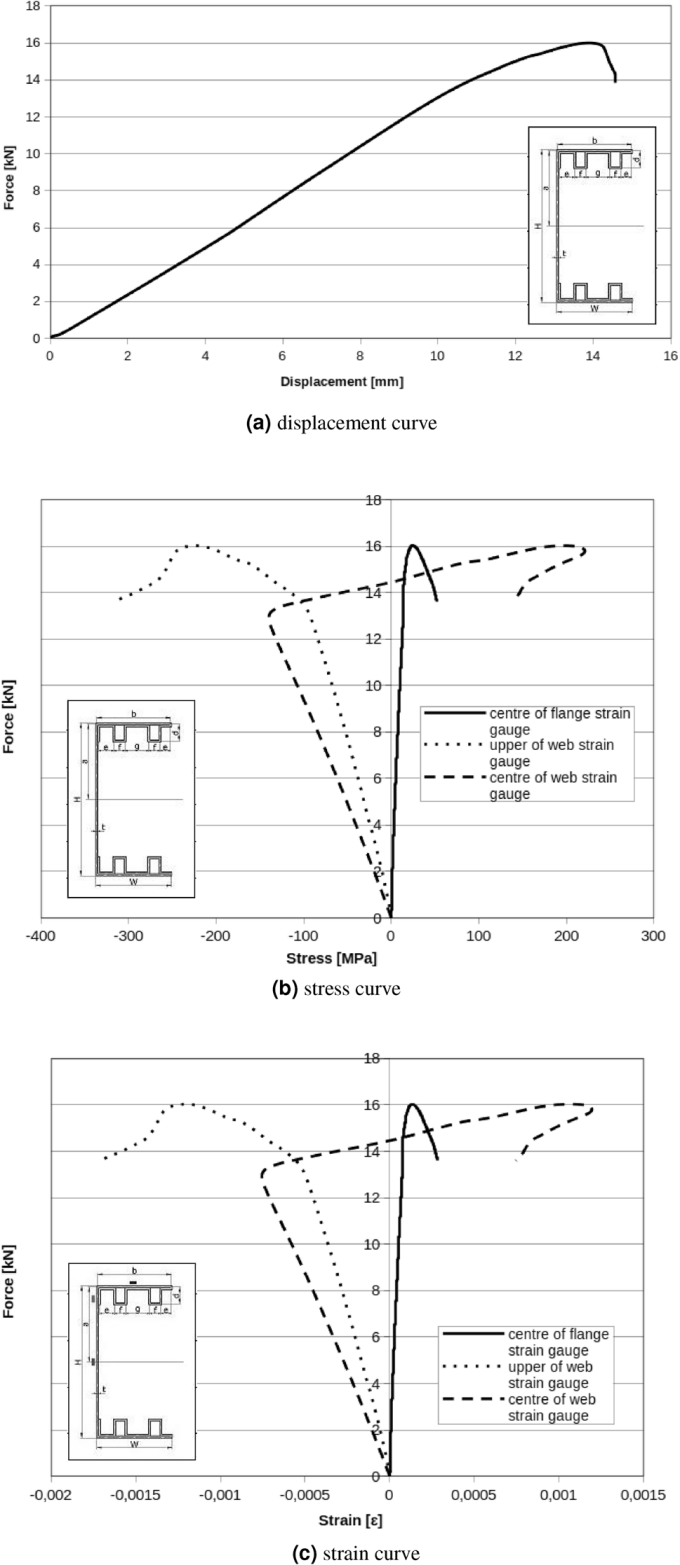
Double box flanges and reinforcement member.

Web displacement points for double box flanges and reinforcement parts also show relative rotation of cross section but now rotation for all measuring points are in the same direction (Fig. 12). Even if setting points and load points are applied in the shear centre, it is impossible to reduce rotation completely. Boundary conditions and applied special handles have influence on observed cross section rotation phenomena. Found critical forces for local and global buckling of double box flanges and reinforcement member respectively are equal: $F_{Lcr}=13\ {\text{kN}}$, $F_{Gcr}=16\ {\text{kN}}$.

**Figure 13:**
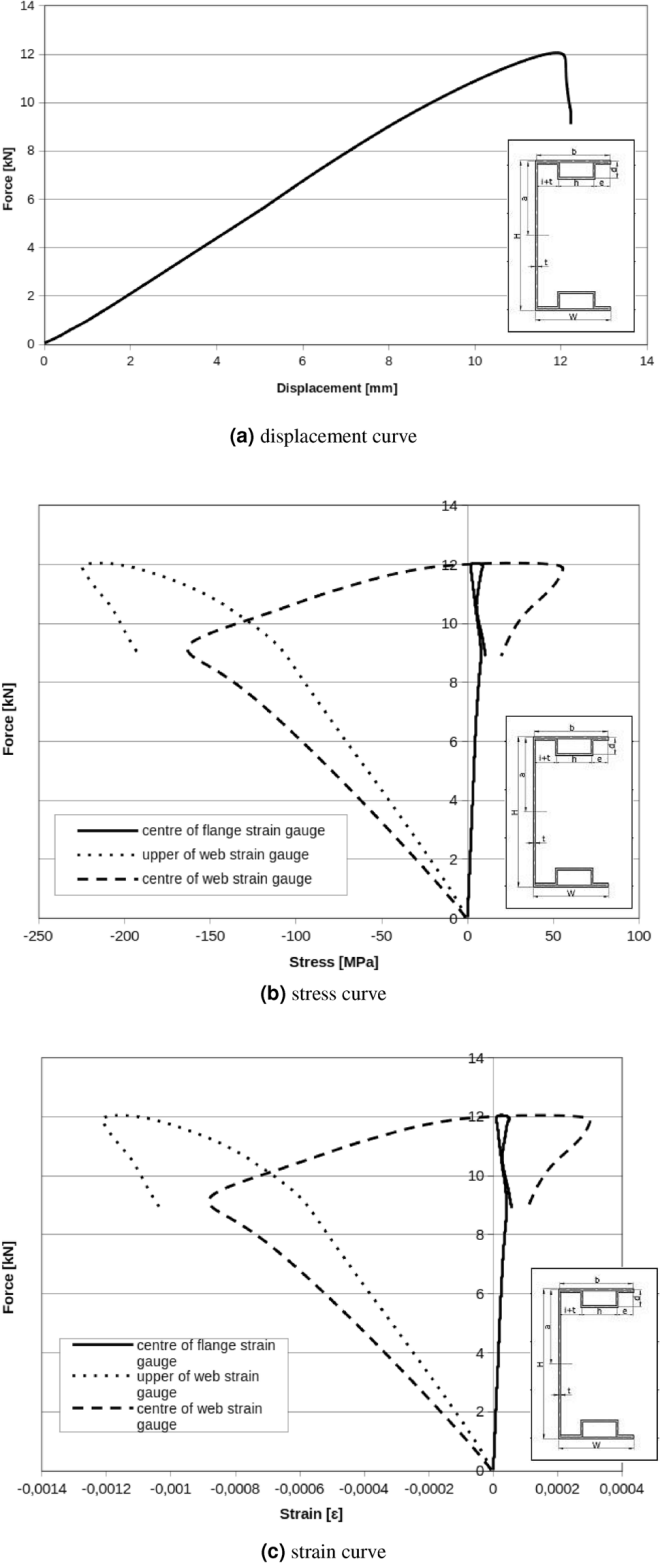
One box flanges without reinforcement member.

Experimental results presented in Fig. 13 for one box flanges without reinforcement parts have lower value of critical forces than double box flanges and reinforcement parts. In this case, critical force for local and global buckling are respectively: $F_{Lcr}=8\ {\text{kN}}$ and $F_{Gcr}=9\ {\text{kN}}$. Displacement results in Fig. 11c show that the rotation of the cross section is registered. For all tested beams it can be observed that subsequent modes of buckling process: local buckling caused by bending moment, distortional buckling phase by bending moment loads and global buckling.

For beams of cross section defined as double box flanges and reinforcement and one box flange without reinforcement, carried out the same tests, where the set and the loads were in the shear centre point of the beams but additionally applied diaphragm such as shown on Fig. 5b.

**Figure 14:**
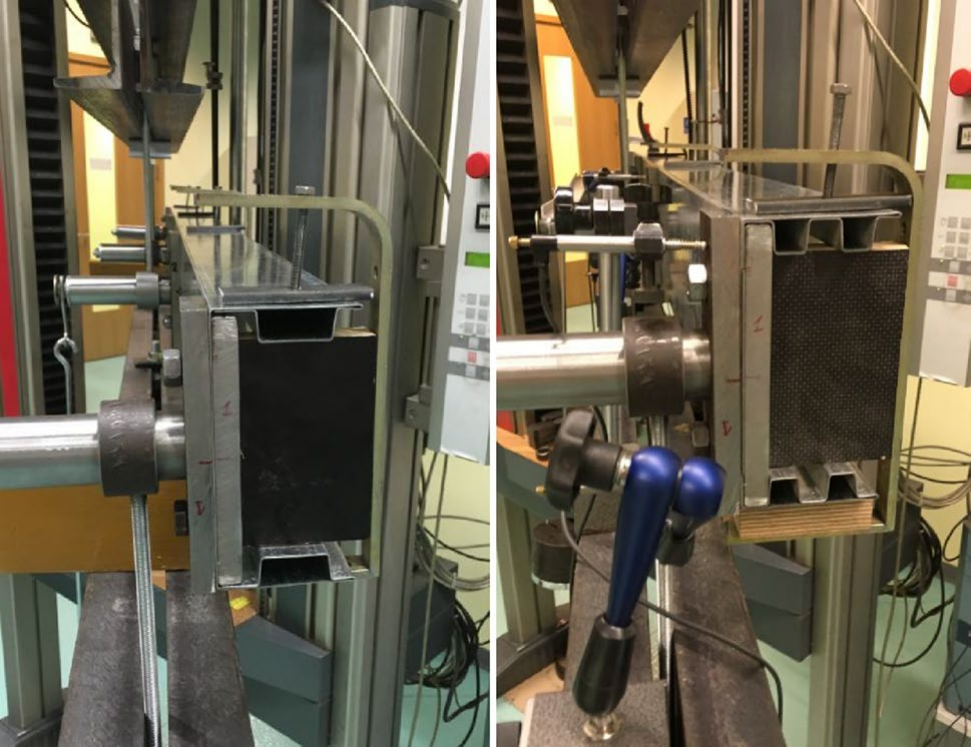
Tested members with plywood diaphragms.

Experimental results for a test, where diaphragms in the handles were applied are presented in Fig. 14. Diaphragms were just plywood reinforcement plate matching to cross section beam shape. Tests with diaphragms were performed for two members: double box flanges with reinforcement beam that is shown in Fig. 4b, and one box flanges without reinforcement beam that is shown in Fig. 4c.

**Figure 15:**
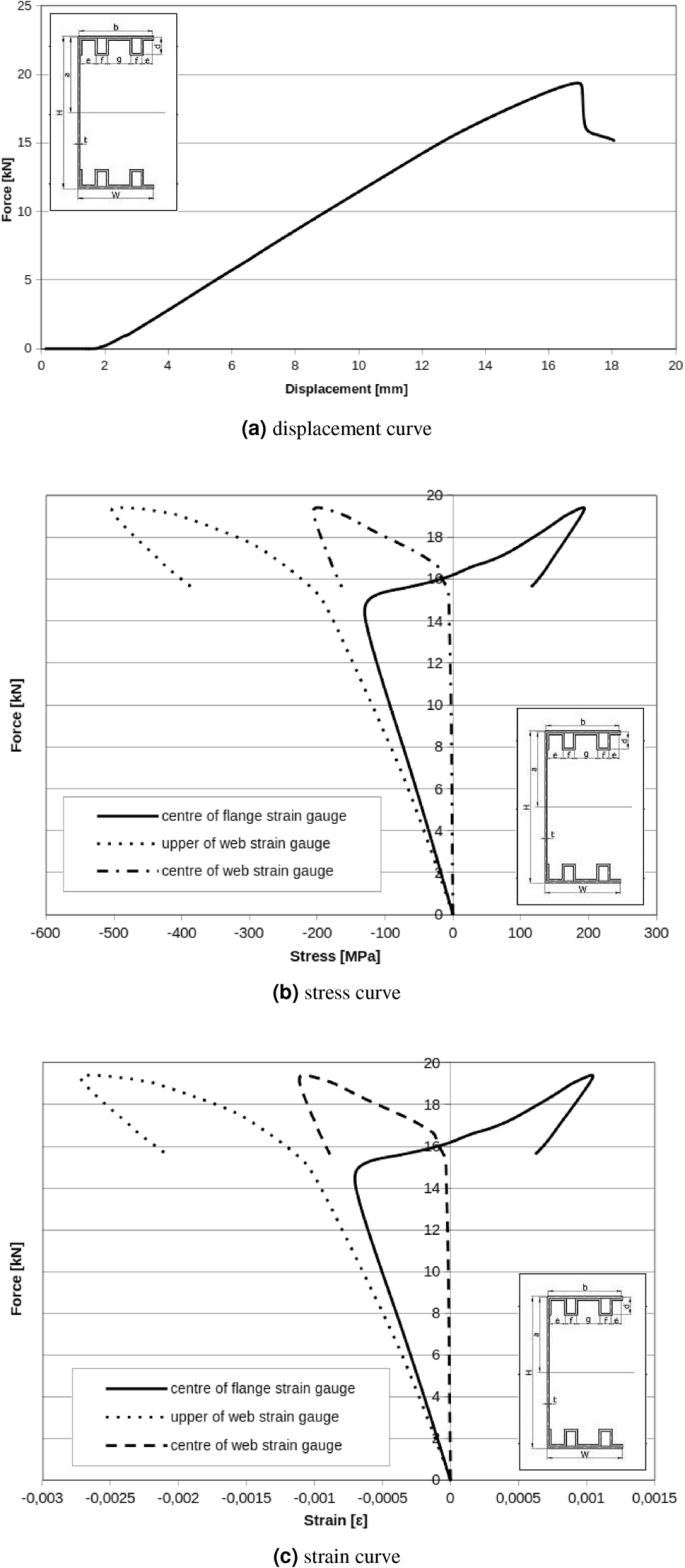
Double box flanges and reinforcement parts with diaphragms.

**Figure 16:**
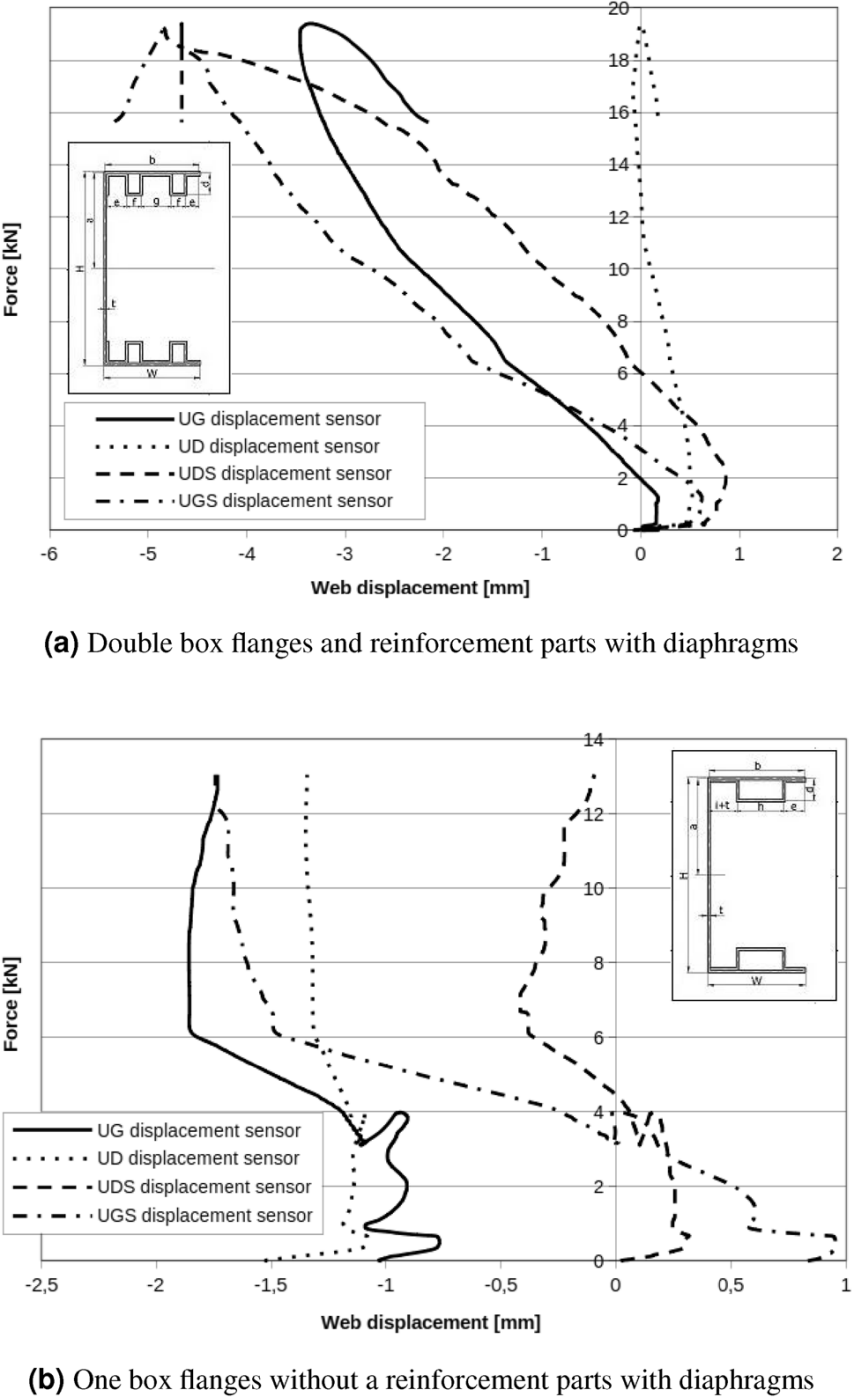
Web displacement points. UG—displacement of the upper web point on the fixed stiff steel plate, UD—displacement of the lower web point on the fixed stiff steel plate, UGS— displacement of the upper web point on the movable stiff steel plate, UDS—displacement of the lower web point on the movable stiff steel plate.

Apply diaphragms as described above, resulted in critical forces for a local and the global buckling respectively: $F_{Lcr}=15\ {\text{kN}}$, $F_{Gcr}=19\ {\text{kN}}$ for double box flanges and reinforcement parts with diaphragms (Fig. 15). Displacement (Fig. 16) measured in the up and the down of handle show a little rotation of beam during test but character of rotation is different than the same beam without diaphragms.

**Figure 17:**
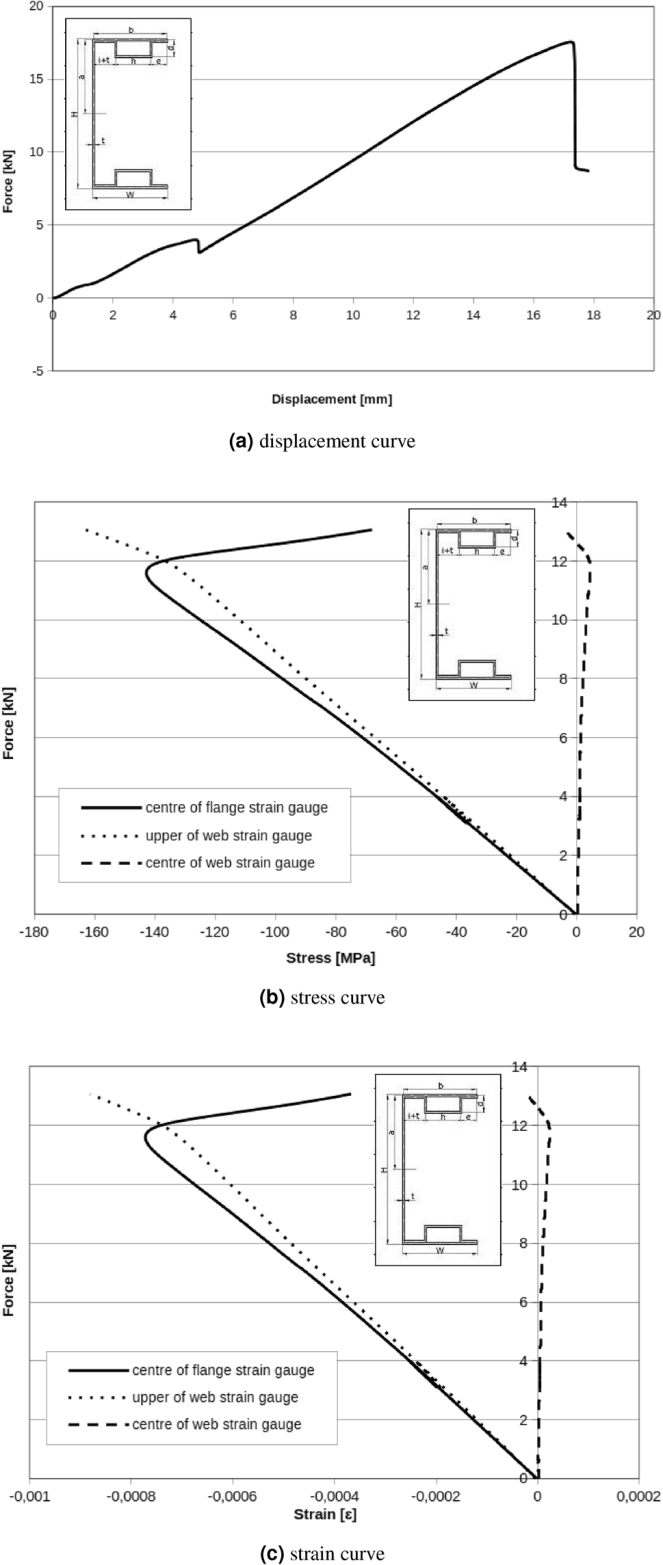
One box flanges without a reinforcement parts with diaphragms.

One box flanges without reinforcement member with applied diaphragms resulted in critical forces for local and global buckling respectively: $F_{Lcr}=4\ {\text{kN}}$, $F_{Gcr}=11\ {\text{kN}}$ (Fig. 17). In Fig. 17a is exactly visible chart peak near $F_{peak}=4\ {\text{kN}}$. It is probably caused by the slip in the handle, setting system or ties. Above $F_{peak}=4\ {\text{kN}}$, curve rise to $F=16\ {\text{kN}}$ and this force can be accepted as global maximal buckling force.

**Figure 18:**
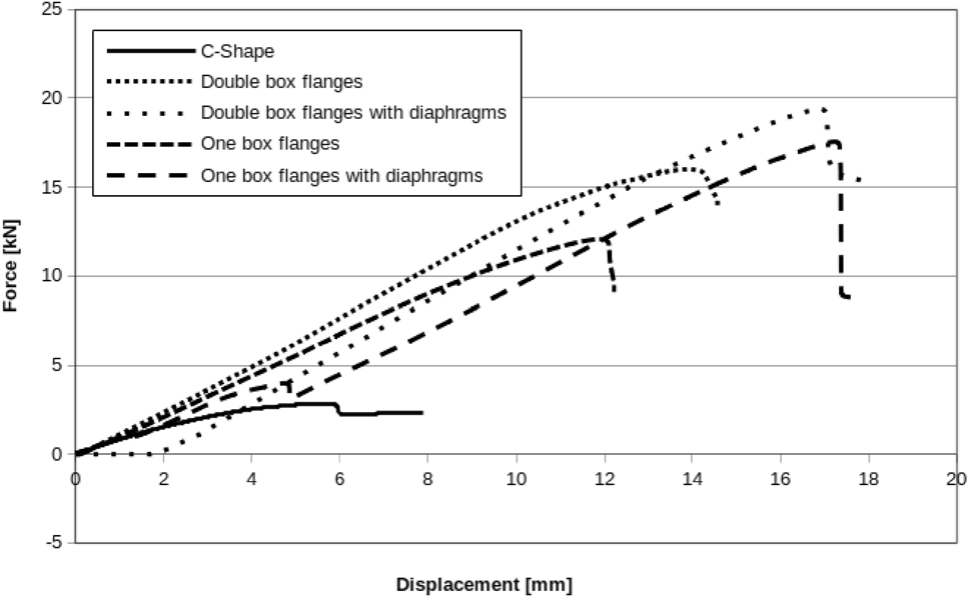
Displacement curve: all beams.

As it’s shown on the Fig. 18, comparing investigated beams load capacity, applying diaphragms, beams are more resisted for buckling. Of course diaphragms are in practical use as stiffness rising elements of thin walled structures, but in the presented research case tried to get pure bending during experimental test. Wasn’t possible to reduce beam cross section rotations perfectly what was caused by imperfect of boundary conditions and beams production process. An additional practical conclusion also arises. Beams accuracy, assembly and exploitation conditions are of key importance for the work of thin-walled systems.

## Discussion

An experimental study of cold-formed thin-walled beams of different cross-sectional shapes, subjected to four-point static bending, with load forcing and support at the shear centre of each beam, allowed the following conclusions to be drawn:

- Thin-walled beams with complex flanges have higher critical forces than thin-walled beams with flat flanges,
- The local loss of stability of thin-walled beams was clearly visible when flanges and webs were observed. Characteristic half-waves have appeared.
- Measurements of web displacements (relative displacements estimated on the basis of readings from the displacement sensors) showed that despite supporting the beams and forcing at the centre of transverse forces, each of the beams rotated around the longitudinal axis passing through the centre of transverse forces of the tested beam cross-section,
- The reason for the occurrence of a torsional moment with a vector directed along the axis passing through the centre of the transverse forces when the beams were supported at the centre of the transverse forces and the load at the centre of the transverse forces was the inaccuracy of the fabrication of the tested beams. The torsional stiffness of the open thin-walled sections is very low, which, with the unavoidable inaccuracies in the fabrication of the beams, results in a torsional moment despite the theoretical action of pure bending between the centre brackets for four-point bending.
- An additional difficulty observed during the experimental tests was the force of gravity which, despite attempts at balancing, influenced the results obtained and the action of torsion moment with respect to the axis passing through the support and load points,
- Testing of sheared thin-walled sections must be carried out with the aid of stiffening diaphragms, especially important when carrying out dynamic tests on open thin-walled beams. Lack of diaphragms results in local failure of the beams and propagation of failure due to local plastic deformation,
- Thin-walled open steel sections are widely used in the construction of road infrastructure, e.g. as energy absorbing panels for road barriers. Appropriate shaping of the cross-section, choice of material and support point are the key guidelines to achieve a high degree of energy absorption during vehicle run-up,
- For energy-absorbing structures, testing on real objects is essential. Thin-walled structures, such as open-section beams, are characterised by high torsional compliance, which is clearly influenced by the accuracy of the thin-walled structure, including the mounting system.

The authors of the research, further work is directed towards issues related to energy consumption of materials and structures. A dynamic test rig has been developed and the results obtained will be presented next.
